# Using Active and Passive Smartphone Data to Enhance Adolescents’ Emotional Awareness in Forensic Outpatient Setting: A Qualitative Feasibility and Usability Study

**DOI:** 10.2196/53613

**Published:** 2024-12-30

**Authors:** Merel M L Leijse, Levi van Dam, Tijs Jambroes, Amber Timmerman, Arne Popma

**Affiliations:** 1 Child and Adolescent Psychiatry & Psychosocial Care Amsterdam UMC location Vrije Universiteit Amsterdam Amsterdam Netherlands; 2 Levvel Academic Center for Child and Adolescent Psychiatry Amsterdam Netherlands; 3 Mental Health Amsterdam Public Health Amsterdam Netherlands; 4 Garage2020 Dutch innovation network for societal youth challenges Amsterdam Netherlands; 5 Faculty of Social and Behavioral Sciences University of Amsterdam Amsterdam Netherlands; 6 iHub Rotterdam Netherlands

**Keywords:** emotion regulation, emotion awareness, smartphone data, forensic outpatient youth care, treatment motivation, treatment alliance, emotion, behavioral, interview, mHealth, app, forensic, usability, feasibility, delinquent, pediatrics, youth, adolescent, teenager, experience, attitude, opinion, perception, perspective, acceptance, emoji, behavioral data, mobile phone

## Abstract

**Background:**

Delinquent behavior in adolescence is a prevalent issue, often associated with difficulties across multiple life domains, which in turn perpetuates negative life outcomes. While current treatment programs show partial success in improving behavioral changes and reducing recidivism, comprehensive conclusions regarding the overall efficacy of these interventions have yet to be established. In forensic outpatient settings, the discrepancy between adolescents’ limited emotional awareness and the predominant emphasis on cognitive reflection, combined with low treatment adherence, may be factors that undermine treatment efficacy. New technologies, such as smartphone apps, may offer a solution by integrating real-life data into treatment to improve emotional and behavioral patterns. The low-threshold use of smartphone data can be useful in addressing these treatment challenges.

**Objective:**

This study aimed to explore the feasibility and usability of Feelee (Garage2020), a smartphone app that integrates active emoji and passive behavioral data, as a potential addition to treatment for adolescents in a forensic outpatient setting.

**Methods:**

We conducted a prepilot study with adolescents (n=4) who used the Feelee app over a 2-week period. App usage included completing a brief emoji survey 3 times a day (active data) and allowing Feelee to track the call logs, Bluetooth devices in proximity, cell tower IDs, app usage, and phone status (passive data). During treatment sessions, both adolescents and clinicians reviewed and discussed the active and passive data. Semistructured interviews were conducted with adolescents and clinicians (n=7) to gather experiences and feedback on the feasibility and usability of incorporating smartphone data into treatment.

**Results:**

The study showed that adolescents (n*=*3) succeeded in using Feelee for the full 2 weeks, and data were available for discussion in at least 1 session per participant. Both adolescents and clinicians (n=7) stated that Feelee was valuable for viewing, discussing, and gaining insight into their emotions, which facilitated targeted actions based on the Feelee data. However, neither adolescents nor clinicians reported increased engagement in treatment as a result of using Feelee. Despite technical issues, overall feedback on the Feelee app, in addition to treatment, was positive (n=7). However, further improvements are needed to address the high battery consumption and the inaccuracies in the accelerometer.

**Conclusions:**

This qualitative study provides an in-depth understanding of the potential benefits of integrating active and passive smartphone data for adolescents in a forensic outpatient setting. Feelee appears to contribute to a better understanding of emotions and behaviors, suggesting its potential value in enhancing emotional awareness in treatment. Further research is needed to assess Feelee’s clinical effectiveness and explore how it enhances emotional awareness. Recommendations from adolescents and clinicians emphasize the need for prepilot studies to address user issues, guiding technical improvements and future research in forensic outpatient settings.

## Introduction

### Background

Delinquent behavior is a prevalent problem, particularly during adolescence, that can be characterized by deviant, violent (or nonviolent), and disobedient conduct [1,2]. The occurrence of delinquent behavior is associated with various risk factors across multiple life domains, such as conflicts at home, peer pressure, and difficulties at school [3,4]. Severe uninhibited delinquent behavior may precipitate additional negative consequences, including school dropout, diminished social support, and a higher likelihood of developing psychiatric conditions [5-7]. Consequently, addressing the underlying multifaceted problems contributing to delinquent behavior often requires prolonged and complex care pathways, which entail substantial social and economic costs [8,9]. Therefore, the individual and societal impacts underscore the critical need for early, targeted interventions to prevent the escalation of multiproblem scenarios and potential victimization [10,11].

Current interventions for adolescents with delinquent behavior are commonly provided in forensic outpatient settings [12,13]. Most treatment programs include elements of cognitive behavioral therapy (CBT) to help adolescent recognize and change thoughts and behaviors that contribute to their delinquent behavior [14,15]. While these are well-established treatment program studies, they report mixed findings regarding the effectiveness [15,16]. For example, a meta-analysis examining CBT treatment outcomes for anger-related problems in children and adolescents, including 21 published and 19 unpublished studies, did not find a convincing overall effect and varying moderate effects among the different components (eg, affective education and problem solving) regarding the CBT intervention [16]. Another meta-analysis of 6 studies reported a significant effect of individual-focused CBT programs for adolescents with aggressive behavior. However, this study also emphasized that only a limited number of individually tailored CBT programs have been developed and evaluated, highlighting the need for more personalized interventions for adolescents [14].

The limited effectiveness of current interventions may be attributable to the emotional abilities of the delinquent adolescent population [17-19]. Adolescents, particularly in forensic settings, frequently encounter difficulties in gaining insight into their emotional and behavioral functioning, which impairs their ability to recognize and express emotions [20-22]. This poses a significant issue, as most treatment programs expect adolescents to develop cognitive control over their emotional responses, which requires them to interpret and reflect on emotional signals to achieve behavioral regulation [23,24]. Consequently, the impaired ability to recognize and express emotions further complicates their capacity to reflect on emotional signs and thus get insight into their own behavioral functioning [25]. This incongruence suggests that existing treatment approaches may not be well-suited to adolescents’ actual capabilities for emotion regulation [22,25].

Another complicated factor in the treatment of adolescent with delinquent behavior is their insufficient engagement with treatment [26]. Adolescents in a forensic setting often show distrust and lack of motivation towards treatment [26,27], which may be attributable to previous failed counseling programs and traumatic life experiences [28,29]. This lack of engagement adversely impacts the therapeutic alliance, as the adolescent’s diminished motivation and trust can compromise the working relationship between the adolescent and the clinician [30,31]. Consequently, treatments in forensic outpatient youth care are plagued by high dropout and incidence of recidivism, estimated between 40% to 50% [30]. Therefore, it is crucial to search for new methods that better encompass the unique needs of adolescents to enhance the efficacy of current therapeutic approaches in forensic outpatient care.

New technologies, such as virtual reality [32], wearables [33], and smartphone apps [34], demonstrate significant potential to address current challenges in treatment. By using these technological applications alongside traditional clinical treatments, clinicians can engage adolescents in a more low-key way [35,36] and gain insight into situations adolescents encounter outside the treatment room [37,38]. These tools generate new data on emotions and behavior in real-life situations, which enrich the treatment setting by offering valuable insights that could improve adolescents’ understanding of the relationship between emotional functioning and behavior [34,38-40].

In particular, smartphone apps that collect both active and passive data could be valuable in current treatment programs [41]. Active data are collected through user actions, typically through a short questionnaire or emotion logbook [41,42]. In contrast, passive data require no user input and are gathered through smartphone sensors, such as a GPS, Wi-Fi–network detection, and a pedometer [41,43]. Although there are various applications of active and passive data, one of the most recognized methods involves tracking emotions and behavior [38,44]. Using both types of data in treatment facilitates regular monitoring of emotions and behavior, which provides a better understanding of the adolescents’ emotional state [38]. In addition, incorporating emojis as a form of active data can introduce an extra dimension, as research has shown that emojis are effective indicators of feelings and individual emotional patterns, which can be valuable in clinical treatment [41,45].

The use of active emojis and passive behavioral data has proven feasible for enhancing self-awareness and autonomy among a general and mental health care population [41]. Given the current challenges in treatments in forensic outpatient care, adolescents in these settings could benefit from it as well [35]. Specifically, integrating emoji and passive behavioral data may be particularly valuable for adolescents struggling to understand their emotions that precede dysregulated and delinquent behaviors [19,39]. Research indicates that using both active and passive data improves adolescents’ self-insight into their emotions and behavior patterns, which in turn enhances emotional awareness [42,46]. In addition, the objectively obtained data foster better self-management among adolescents, which potentially increases their engagement in treatment [47].

### Objectives

In this study, we conducted a prepilot to explore the feasibility and usability of a new smartphone app, Feelee (Garage2020), designed to collect and display both active and passive smartphone data for use in forensic outpatient treatments. After a 2-week test period, we conducted semistructured interviews with both adolescents and clinicians to gather feedback and insights regarding their experiences with the app and the integration of active and passive data into treatment. The interviews specifically focused on evaluating the potential of this new intervention to enhance adolescents’ emotional awareness and engagement with their treatment.

## Methods

### Overview

To gain insight into experiences regarding the feasibility and usability of the Feelee app in a forensic outpatient setting, we conducted a prepilot study. Participants first tested Feelee alongside their ongoing treatment. Subsequently, we collected semistructured interviews with the participants and their clinicians.

### Participants

Clinicians from the youth care organization “Levvel” in Amsterdam initiated this research project by a foundation called “onzeHOOFDzaak,” aimed at conducting innovative research to improve treatments for youth in forensic settings. The forensic outpatient services at Levvel provide treatment for adolescents (aged 13-23 years) and their families, often under mandatory conditions as part of a conditional sentence. Participant recruitment took place between October 2020 and June 2021. Given the challenges of engaging a forensic population in research, clinicians were closely involved in both recruitment and data collection. This lead researcher and clinicians initially selected potential participants based on 2 eligibility criteria: (1) adolescents had to be receiving treatment at the forensic outpatient team of Levvel during the study, and (2) they had to own an Android phone, as Feelee only operated on android operating systems. Exclusion criteria included (1) adolescents not maintaining sufficient contact with their clinician and (2) those with insufficient proficiency in Dutch. Recruitment concluded when data saturation was reached [48].

### Procedure

Considering the importance of end-user engagement in developing effective technical applications [49,50], we determined the involvement of adolescents and clinicians as a crucial element of this feasibility and usability study. To address this, the executive researcher organized two workshop sessions to (1) introduce clinicians to the Feelee app and (2) involve clinicians in shaping this study and discuss the app’s clinical implementation. Between the workshop sessions, clinicians independently used Feelee for 2 weeks. All 12 clinicians from the forensic outpatient team participated during the workshop sessions, including a psychiatrist, psychologists, family therapists, and a social worker. We used their feedback to develop the topic list for the semistructured interviews at the end of the adolescents’ Feelee test period.

Recruitment started with clinicians reaching out to adolescents from their caseloads. Adolescents who met the inclusion and exclusion criteria and consented to participate were invited by the research team for an initial briefing about the study. After a minimum of 7 days, informed consent was obtained, allowing adolescents to participate in the study. From then, adolescents started using the Feelee for a minimum of 2 consecutive weeks, selecting an emoji at 3 different times each day (preferably morning, afternoon, and evening). In weekly treatment sessions, adolescents and clinicians reviewed and discussed the active and passive data from Feelee, which were presented in a dashboard. The executive researcher observed these sessions and took notes for the logbook, which we later used to inform the semistructured interviews. We conducted separate face-to-face semistructured interviews with both adolescents and clinicians. In total, 2 distinct topic lists were conducted to explore their attitudes and perspectives on the app’s feasibility and usability. A user experience designer with expertise in feasibility and usability studies reviewed these lists, and we validated the constructs against existing scientific literature to ensure their robustness. The topic list covered themes such as general experiences with Feelee, emotional and behavioral aspects, treatment motivation, treatment alliance, and ideas to improve Feelee. The interviews and topic lists were tested through role-play before the study. Each interview lasted 30-45 minutes.

### Feelee App

**Figure 1:**
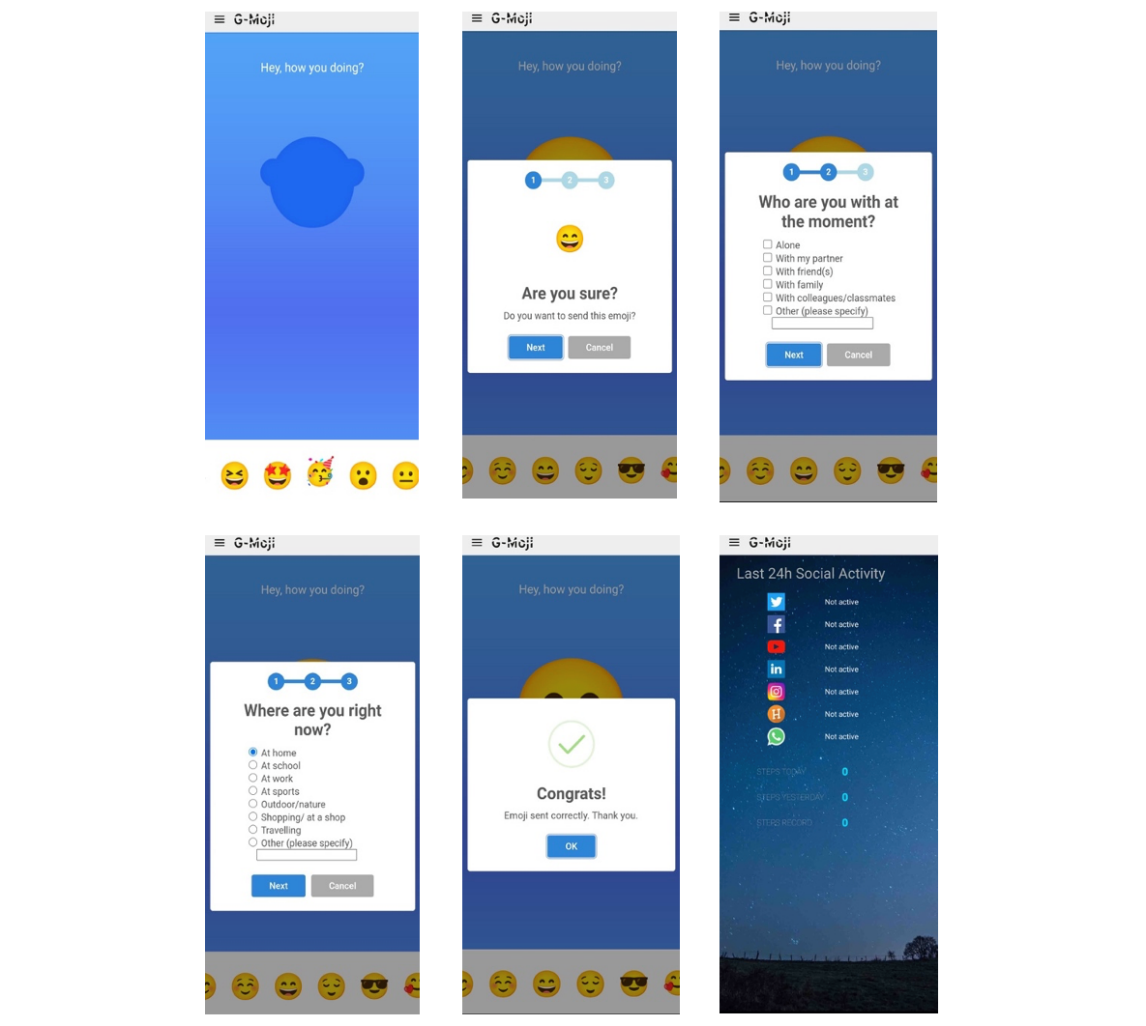
Screenshots of the Feelee app used by adolescents during the test period: main screens.

**Figure 2:**
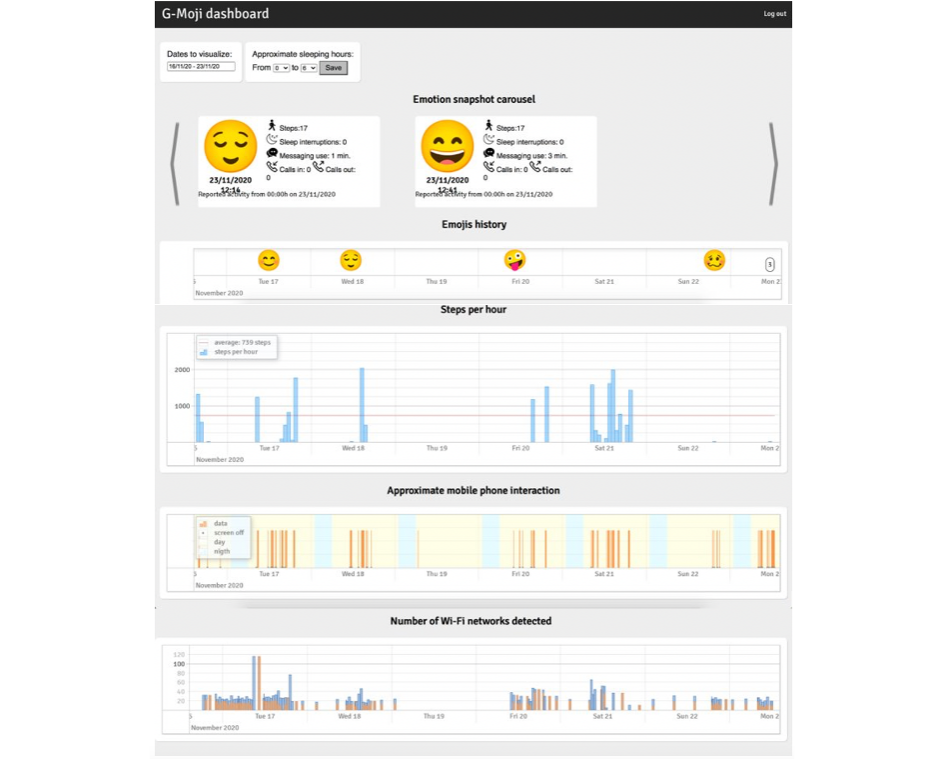
Screenshot of the external dashboard of the Feelee app used by adolescents and clinicians during the test period.

Feelee is a product of Garage2020, which is an innovation network for youth care in the Netherlands. Feelee collects and displays both emoji’s (active) and behavioral (passive) data. Users provide emoji data by submitting an emoji and answering a few additional questions. For this study, we used a research version of Feelee called G-Moji, which was compatible only with Android operating systems. The app operates as follows: it sends notifications 5 times a day (9 AM, 11 AM, 1 PM, 5 PM, and 7 PM) asking, “Hey, how are you doing?” Users can submit an emoji at any time during the day and without any limitations. Once an emoji is submitted, Feelee prompts users with follow-up questions, such as “Are you sure?” “Who are you with at the moment?” and “Where are you right now?”(more details in Figure 1). In addition to emoji data, Feelee collects passive data, including call logs, Bluetooth devices in proximity, cell tower IDs, app usage, and phone status (eg, charging and idle). Passive data are also displayed in an external dashboard, which includes metrics such as the number of steps per hour, approximate mobile phone interactions per hour, number of call minutes, and the number of detected Wi-Fi networks (more details in Figure 2). Users can access this external dashboard through an internet browser using a log-in and a linking code provided in the Feelee app. The Feelee data were used solely for treatment purposes during the 2-week test period, not for conducting further study analyses.

### Data Analysis

All interviews were recorded and transcribed anonymously. The 7 transcripts were then compiled into a single table in Microsoft Word for analyses by 2 independent analysts. The table included three separate columns: (1) codes, (2) transcripts, and (3) notes. The data analyses occurred in two phases: (1) open coding and (2) thematic coding. In the open-coding phase, the first author began with an in-depth reading of the transcripts and assigning initial codes. Then, the first author sorted, interrelated, and grouped the open codes based on inductive analysis [51]. The fourth author followed the same process. Subsequently, both analysts compared their codes, discussing any differences, additions, and uncertainties. In the same session, the analysts identified themes to initiate the thematic coding phase. During this phase, the first author reread the transcripts to assign codes to identified themes using a data matrix [52]. The fourth author reviewed the thematic codes in the data matrix, and any differences, additions, and doubts were addressed through further discussion. Throughout both the open coding and thematic coding phases, the analysts maintained a logbook to document their steps, notable observations, and the justification for specific analytical decisions.

### Ethical Considerations

The study received approval from the Ethical Review Board of the University of Amsterdam, the Netherlands (ERB: 2021-CDE-13174). Informed consent was required and obtained from all participants. No individuals under the age of 16 years were enrolled in this study. During informed consent, adolescents gave explicit permission for the audio recording of interviews and for the collection of both active and passive data by the Feelee app. The audio recordings were used to generate deidentified transcripts of the interviews and were subsequently destroyed immediately after transcription. Both the audio recordings and transcripts were securely stored within the research department on a protected hard drive. Data collected from the Feelee app were aimed at gather experiences from adolescents and clinicians in the usage of active and passive smartphone data. These data were stored anonymously on a secure server in compliance with the General Data Protection Regulation (GDPR). Furthermore, the Feelee data were not used for any other research purposes. This manuscript does not contain any identifiable images or data related to human subjects. Participants received a gift voucher (26.05 USD) for their participation.

## Results

### Participants Characteristics

During the Feelee test period, adolescents (n=4) participated in this study alongside their ongoing treatment at the forensic outpatient setting. All participating adolescents were male (4/4, 100%), which reflects the predominantly male demographic of the forensic population, though our sample slightly overrepresents this group relative to the broader population [53]. The mean age of participants was 18.5 (SD 0.58) years. Most adolescents (3/4, 75%) were engaged in individual treatments, including psycho-education, CBT, or trauma treatment. Following the test period, interviews were conducted with both participating adolescents *(*n*=*4) and involved clinicians (n=3). Furthermore, 1 clinician was involved in the treatment of 2 participating adolescents, and one of the clinicians used Feelee voluntarily for themselves during the test period.

### Feelee Test Period

#### Adolescents’ App Usage

Adolescents in this study used their personal smartphones. However, 1 adolescent who owned an iPhone arranged to use an Android smartphone. Logbook data indicated that all adolescents successfully used Feelee for 2 consecutive weeks except for one who used it intermittently over a period of 3 months. The frequency of Feelee usage varied among participants. One adolescent consistently logged an emoji 3 times a day throughout during the 2-week test period. In addition, two others used Feelee for 2 weeks but did not meet the goal of logging an emoji 3 times a day. However, they did manage to log at least 1 emoji per day for 75% of the test period. The adolescent who used Feelee for 3 months on and off was mostly successful in logging an emoji 3 times a day. During the interviews, when adolescents were asked about the frequency of logging emojis 3 times a day, half (2/2, 50%) of the adolescents found this frequency acceptable, while others preferred to log only 1 emoji per day. In terms of passive behavioral data, 75% (3/4) of the adolescents allowed Feelee to collect the passive data. One adolescent had disabled this feature, so no passive data were available.

#### Treatment Characteristics

In addition to using the Feelee app, adolescents discussed both their active emoji and passive behavioral data with their clinicians during weekly treatment sessions through the external dashboard. For one participant, the external dashboard malfunctioned. With another adolescent, the clinicians successfully discussed the active and passive data twice during the 2-week test period. The data were discussed once for the remaining 2 adolescents. Regardless of the clinician involved, the process followed a similar pattern: first, the adolescent and clinician reviewed all available active and passive data on the external dashboard. Then, the clinicians initiated clarifying or reflective questions about notable details in the data. These questions varied depending on the adolescent: for one, the discussion focused on emotions from the previous week, along with the corresponding passive behavioral data. For others, the emphasis was on the passive data, such as sleep patterns and pedometer readings.

### Interviews

#### General Experiences With Feelee

Based on the interviews, both adolescents and clinicians expressed general enthusiasm about Feelee. All adolescents (n=4) described the app as “nice” and valuable to use in addition to their treatment. Clinicians (n=3) were also pleasantly surprised by Feelee, noting that, despite its simplicity, the app had a significant impact in a short period of time:

It did surprise me because...I didn’t expect it to do so well. And also, that you could quickly get something out of it to use for the session again.

Clinician 3

However, both adolescents and clinicians also expressed criticism regarding Feelee’s functionality due to several flaws. The most commonly mentioned (n*=*3) was the apps’ high consumption. Due to the passive data collection, the smartphone battery drained significantly faster than usual. In addition, the pedometer proved to be inadequate. According to 1 adolescent, the recorded steps did not match their actual activity levels, leading to an inaccurate representation of their physical activities. Finally, for 1 adolescent, the external dashboard failed to function properly.

In relation to Feelee’s functionalities, adolescents had some feedback regarding the amount of data collected, especially passive data collection. Most expressed that they had no issue with Feelee collecting personal data. For example:

You can go left, you can go right, but your data is collected anyway. So, personally, I don’t have a problem with it.

Adolescent 2

Regarding the data collection, 1 adolescent admitted being “shocked” by the amount of data displayed in the external dashboard, especially the number of messages and call minutes. However, the adolescent declared to be “delighted” that there were no further details visible. In addition to that, the other adolescents (n=3) mentioned that they had no issues with the data being collected. In addition, 1 adolescent expressed to find it convenient to receive personal information in this way. However, another adolescent raised concerns about the collection of personal data, stating it is acceptable as long as it remains superficial for users, with clear explanations provided upfront about what data are being collected and how they will be displayed in the app.

#### Feelee Usage in Treatment and Its Impact on the Adolescents’ Emotions and Behavior

Following the general experiences, adolescents and clinicians were asked to provide specific feedback on the usage of the Feelee app in treatment. The data analysis revealed four distinct steps that describe the process of using Feelee in treatment and its impacts on adolescents’ emotions and behavior: (1) viewing, (2) discussing, (3) insight, and (4) action. Each of these 4 steps is discussed in detail below from both the adolescent’s and clinician’s perspectives, with an overview provided in Table 1.

**Table 1:** Overview of themes and statements from both adolescents and clinicians regarding the use of Feelee in treatment.

Steps Feelee in treatment	Adolescents (n=4)	Clinicians (n=3)
Viewing	Found it interesting to see the data (n=2) Feelee is helpful to think for a moment about emotions and relate to them (n=4)	Feelee provided new information about the adolescent’s behavior (n=2) Feelee makes emotions and behavior more tangible and less abstract for adolescents (n=3) Feelee supports adolescents to view their emotions in a more secure way (n=3)
Discussing	Initially hesitant to share the Feelee data with the clinical worker (n=4) Conversation with the clinician about the Feelee data was pleasant (n=2)	Feelee is helpful when starting a conversation with adolescents since they pretend nothing is happening (n=3) Feelee supports a low-key conversation: it helps to recall emotions and behavior from the past (week) (n=3)
Insight	Feelee is valuable in gaining more awareness about emotions (n=3) Insights resulting in: New insights about emotions (n=3) A more in-depth understanding about existing issues of the adolescents (n=2) Feelee supports making a connection between emotions and the influence of their environment and (daily) activities (n=3)	Feelee is helpful in providing adolescents with more insights into their emotions because they avoid it too easily (n=3) The Feelee data offers different perspectives on adolescents’ emotions and linking behavior (n=2) Feelee helps to link emotions with behavior, which creates more awareness among adolescents (n=3)
Action	Insights from the Feelee data led to an action based on the experienced emotions (eg, going for a bike ride or walk) to calm down (n=2)	Insights from the Feelee data resulted in a more specific focus on the adolescents’ sleep disturbance issues in the treatment (n=2)

#### Viewing

The first step, “viewing,” involves analyzing the active emoji data and passive behavioral data in the Feelee app and external dashboard, both individually by the adolescent and in collaboration with the clinician. Overall, adolescents (n=2) expressed that they were “curious” and found it “interesting” to see the data. In terms of its impact on emotions and behavior, all adolescents (n=4) noted that Feelee helped them to think for a moment about their emotions and reflect on them. An adolescent expressed it as follows:

...because it really gives you time to think about it. Normally, you are just grumpy while during your daily activities. And when you're grumpy, you’re just focused on being grumpy, and then something else comes along.

Adolescent 4

For clinicians (n=2), viewing the Feelee data provided new insights into the adolescents’ emotional and behavioral patterns. In one instance, a clinician discovered that the adolescent’s sleep disturbances were more severe than previously anticipated. In another case, the data served to validate a clinicians’ suspicions regarding the adolescent’s excessive screen time, reinforcing concerns about the adolescents’ mobile phone use. Furthermore, clinicians (n=3) found that Feelee not only offered valuable insights from the adolescents’ perspectives but also encouraged the adolescents to take a moment to think about their emotions:

Well, that it’s a lot to gain because normally they don’t think about it, but with the emoji’s they apparently do. And they take it more seriously than when I just tell them.

Clinician 2

As noted by the clinician, adolescents take emotions and related behavior presented in Feelee more seriously. Furthermore, all clinicians (n=3) mentioned that Feelee makes emotions and behavior more tangible and less abstract, enabling adolescents to better comprehend their feelings and actions. Consequently, the Feelee app supports adolescents to become more aware of their emotions and behavior in a safe and supportive way.

#### Discussing

The next step involved “discussing,” which refers to the conversation between the adolescents and the clinicians about the Feelee data. All adolescents (n=4) indicated that they were initially hesitant to share and discuss the Feelee data with the clinician. As an adolescent remarked:

...Things can get confrontational...That’s the feedback I have too: What if I have a really bad week? Am I then obligated to talk about it?”

Adolescent 2

Other reasons adolescents cited included finding it annoying to expect questions, feeling controlled by clinician’s inquiries, not seeing the benefit of discussing emotions, or simply not feeling inclined to talk about them. However, adolescents (n=2) did describe their conversation with the clinician about the Feelee data as “pleasant.” In addition, interviews with clinicians indicated that Feelee encourages adolescents to discuss their emotions. According to clinicians (n=3), adolescents often pretend that nothing is wrong and dismiss their feelings with laughter, yet there is more beneath the surface. Nonetheless, the overview of the Feelee data supports adolescents articulating their emotions and behavior more openly:

Well, I did expect the adolescent to feel angry more often, and to feel stressed and things like that, but I didn’t expect them to talk about it more easily because of the pictures. Normally, the adolescent would just say nothing was going on, but now you could clearly see what was happening.

Clinician 1

More specifically, clinicians (n=3) noticed that the Feelee data create an opportunity for “easier conversation” with the adolescents. This facilitated a more low-key conversation about emotions and their linking behavior:

...Indeed, the adolescent moves very little. But based on that, you can easily discuss it with them, even without actually seeing the numbers. It naturally comes up in the conversation.”

Clinician 2

During the conversation, the Feelee data proved supportive for both adolescents and clinicians in recalling emotions and behavior from the past week. As a clinician explained, a week can feel very long for adolescents, and the overview of the Feelee data helps to identify specific moments for further discussion. This approach makes conversations about emotions and behavior easier for adolescents to understand. Consequently, a clinician mentioned that after the Feelee test period, one of the adolescents became more willing to share their emotions and experiences during treatment sessions.

#### Insight

After discussing, the third step in using the Feelee in treatment involved “insight.” Based on the interviews, insight refers to the process of gaining awareness about emotions, recognizing the connections between these emotions, environmental influences, and the fulfillment of activities. Most adolescents (n=3) expressed that Feelee was valuable in enhancing their awareness of emotions, leading some to new insights. For instance, one adolescent noticed that it was “nice” to see the emojis were generally more positive than expected. For others (n=2), the insight derived from the Feelee data offered a deeper understanding of their existing issues:

Well, I have to say that I’ve had sleep problems for as long as I can remember. Maybe I was in a bit of denial then, too, but I really didn’t realize it was that bad.

Adolescent 2

In addition to fostering awareness, adolescents (n=3) explained that Feelee helps them to establish connections between their emotions and the influences of their environment and (daily) activities. For example, adolescents (n=2) noted that it was “nice” to get insight into how they felt about specific daily activities such as work. For others (n=2), Feelee was useful in understanding how their emotions were affected by certain environments, such as home or socializing with friends:

Your mood is definitely affected...At a certain point, you start to notice that, yes, if I’m with friends, I’m often a bit happier. Or if I’ve just gotten home and relaxing, I’m also happier. But if I’m at home all day, I tend to be a bit grumpier.

Adolescent 4

Clinicians also found Feelee helpful in providing adolescents insight into their emotions and linking those to their behavior. According to clinicians (n=3), adolescents in the forensic outpatient setting often have limited insight into their emotions, and Feelee supports them in gaining that insight in a “quick and effective way”:

Well, they might more quickly realize, “Hey I was a bit more tense back then, I was a bit angry.” This way, some can gain better insight into their feelings. Normally, they tend to brush it off too easily.

Clinician 2

In addition, clinicians (n=3) found Feelee to be supportive in creating greater awareness among adolescents. According to clinicians (n=2), the external dashboard offers adolescents a different perspective on their emotions and behavior compared with discussions with the clinician:

...Well, I think it was really helpful for them to see that, and it helps me, too. I can say, “you should do more in a day instead of just gaming,” because then you might sleep better. When they see the data themselves, it’s not only the stupid psychologist who says it, but it is also on a screen in front of you.

Clinician 3

#### Action

The fourth and final step in using the Feelee app in treatment is “action.” After gaining insight into emotions and behavior, some adolescents (n=2) reported successfully taking steps to improve their emotional well-being. For example:

Sometimes I know I feel like shit right now or something, so I go for a bike ride just to calm down. So yes, I do get something out of it from time to time.

Adolescents 4

Furthermore, for 1 adolescent, insights gained from the Feelee data resulted in new treatment steps focused on addressing sleep disturbance issues more precisely. The clinician involved stated to be surprised by the insights derived from the Feelee data. Since then, greater attention has been given to the adolescents’ sleep disturbance issues during the treatment sessions.

#### Adolescents’ Engagement With Treatment

In addition to examining the impact on adolescents’ emotions and behavior, we also sought to understand their engagement with treatment. To this end, we asked both participants and clinicians about the adolescents’ motivation for treatment and treatment alliance.

Regarding motivation, both adolescents (n=4) and clinicians (n=3) stated that Feelee did not necessarily enhance adolescents’ motivation for treatment. Both indicated that the adolescents participating in the study were already motivated. However, adolescents (n=2) still reported that while they do not particularly enjoy attending treatment, they now perceive it as more helpful than before. Clinicians (n=3) reported that the addition of Feelee would be the most effective when adolescents are more motivated to follow treatment. In addition, 1 clinician noted that Feelee may also be effective at the beginning of the treatment, as it can stimulate adolescents’ curiosity.

Regarding the treatment alliance, adolescents (n=4) and clinicians (n=3) assessed their relationship as positive. All adolescents (n=4) considered the contact with their clinician to be pleasant. Clinicians (n=3) share this view but also note that it often takes a considerable amount of time to develop such a strong treatment alliance, as seen with the current participants. Since both adolescents and clinicians stated that the treatment alliance was already strong, they believe that Feelee did not necessarily enhance it. However, clinicians feel that Feelee could help them to better understand adolescents and establish their connections with them in a more accessible way:

Yes, well, I think...because you discuss it without it being directly about them, you can establish a connection in a very accessible way. I can see how this might help in building the treatment alliance.

Clinician 3

### Suggestions for Future Directions

Based on the use of the Feelee app in the 2-week test period, both adolescents and clinicians provided suggestions for further development. These suggestions can be categorized into 2 main areas: general improvements to Feelee and its application to treatments.

#### Feelee App in General

All adolescents (n=4) expressed interest in using Feelee again, provided that improvements are made in its functionality and design. An adolescent highlighted the importance of having Feelee available on iOS operating smartphones. A common suggestion among the group was that the emojis should convey clearer meanings, as adolescents (n=2) felt the emojis were too open to interpretation. In addition, adolescents (n=2) found the design of the G-Moji app too childish, which made it less appealing. Consequently, adolescents (n=3) indicated that this was a key reason they would not recommend Feelee to their friends.

#### Feelee App as Addition to Treatments

Both adolescents and clinicians offered several suggestions for integrating Feelee into treatments. For instance, 1 adolescent noted that Feelee could help reduce stress by serving as a reminder to manage tasks that might otherwise cause stress. Another adolescent (n=1) suggested using emojis to capture emotions before and after treatment sessions. One of the clinicians highlighted that Feelee could serve as an accessible homework assignment, allowing them to select specific moments from the Feelee data for deeper with the adolescent. This approach makes emotions and behavioral changes more tangible for adolescents, potentially increasing adolescents’ engagement in treatment. However, 1 adolescent and 1 clinician emphasized that Feelee should never feel like an obligation to treatment. According to the clinician (n=1), it should remain noncommittal and approachable. Furthermore, clinicians (n=3) indicated that Feelee would be more usable for adolescents who avoid discussing emotions in treatment. Hence, the use of Feelee in treatment should be tailored to the specific needs of each client and the type of nature of their treatment:

...I think it really depends on the adolescent’s specific problems and where they are in their treatment journey. If they’re not used to talking about emotions and they make an effort to do so by using the G-Moji app, and then discuss it with you, that can be very helpful for building the treatment alliance.

Clinician 3

Regarding the future of Feelee, clinicians (n=2) and a single adolescent (n=1) suggested the idea of remote monitoring. Remote monitoring involves collecting and tracking data on clients’ progress and well-being from a distance. This allows clinicians to send messages less frequently, as they can continuously observe how clients are doing and intervene when necessary. This approach can contribute to the provision of care by enabling timely interventions and offering more tailored support based on real-time data.

## Discussion

### Principal Findings

To our knowledge, this study is among the first to explore the feasibility and usability of incorporating both active and passive smartphone data into ongoing treatments within a forensic outpatient setting. The results from a 2-week test period provide an initial indication that, despite the short duration of this prepilot, the use of both active and passive data is experienced as a valuable addition to adolescents’ treatment. Interviews with adolescents and clinicians revealed generally positive responses to the inclusion of the Feelee app in treatment as usual. Regarding adolescents’ experiences with their emotions and behavior, the data collected by Feelee were found to be useful during treatment sessions across 4 distinct phases: viewing, discussing, gaining insight, and taking specific actions. However, the app was seen as less helpful in boosting adolescents’ motivation for treatment or strengthening the treatment relationship with them and clinicians. In addition, both adolescents and clinicians expressed critical views regarding certain technical issues and the design of the Feelee app, leading to several recommendations for future app development and its potential application in treatments.

Given current challenges in treatments, we explored whether and how the use of active and passive smartphone data could be integrated into forensic clinical practice. Consistent with previous research on adolescents with general mental health issues, the results from the 2-week test period indicate that both adolescents and clinicians found the use of active and passive data alongside treatment sessions to be feasible and valuable [44,54,55]. As anticipated, discussing the data during treatment offered new insights into the adolescents’ daily emotional and behavioral experiences [37,56]. However, the way the Feelee data were addressed in the treatment sessions varied among adolescents. For some, the focus was on emoji data, while for others, it was on passive data. To gain a clearer understanding of how Feelee can be effectively used in treatments, future clinical research should implement a more structured procedure for discussing Feelee data during treatment sessions.

In general, the experience of adolescents and clinicians with Feelee alongside treatment was positive, although there were critical evaluations concerning the app itself. Specifically, users reported that the app placed a substantial drain on their phone batteries, had an inaccurate step counter, and experienced functionality issues with the external dashboard. Such technical challenges are not uncommon in the early stages of app research [57,58] and emphasize the importance of conducting prepilot studies in clinical practice to identify user issues early and incorporate them into the app’s further development [59]. This also applies to the personal data collection in this type of app research, where it is crucial to engage end users in considering the ethical implications involved [60]. While most adolescents in this study did not express objections to the data collection process, their feedback highlighted the necessity for clear communication about what data is collected, how it is collected, and the purposes for which it is used. This finding is consistent with previous research on adolescents’ perspectives regarding smartphone data collection [61], which suggests that while most adolescents are receptive to the collection of mental health data for related purposes, this acceptance is contingent upon principles of transparency, control, and support within the context of personal sensing. Future research involving the collection of both active and passive personal data should address these ethical concerns and incorporate these principles to enhance user trust and engagement [62].

Looking more closely at the use of Feelee during treatment sessions, the semistructured interviews with both adolescents and clinicians provided a deeper understanding of the experiences and attitudes of both. Given that adolescents often face challenges with their emotional awareness, we were particularly interested in their experiences using active and passive smartphone data in treatment to enhance emotional awareness [34,41,55]. The interviews revealed that Feelee the therapeutic process in 4 different key steps: viewing, discussing, insight, and action. As shown in previous studies, the objectively measured active and passive data gave adolescents a more concrete understanding of their emotions and behavior in real-life situations [37,63]. These findings demonstrate that a visual representation of the data from Feelee helped initiate conversations about emotions and related behaviors during treatment sessions. This reflective dialogue is crucial in developing insight into emotions, concerning them to behaviors, and fostering the ability to take further action [20,63]. Consequently, the integration of Feelee into treatment could be valuable in improving adolescents’ emotional awareness, which is a critical first step in enhancing emotion regulation skills [25,39]. However, further research is needed to examine the clinical effectiveness and underlying mechanism of Feelee, for example, through the use of a single-case experimental design.

Regarding treatment engagement, the use of Feelee did not appear to strengthen the adolescents’ motivation for treatment and treatment alliance. This may be attributed to the fact that this study mainly included adolescents who had already been in treatment for an extended period, during which both motivation and treatment alliance had been firmly established. As such, it could be beneficial to examine the effects of Feelee across different phases of treatment, particularly in the initial stages, where adolescents typically demonstrate lower motivation and a more fragile treatment alliance [29,30]. The interviews suggest that Feelee may help build rapport by promoting a more accessible and shared understanding. Furthermore, Feelee could serve as a potential intervention to reengage adolescents when treatment progress stagnates. Regarding adolescents, the integration of active and passive data from the app contributes to improved self-awareness and self-regulation of daily emotional experiences across varying contexts [47,64]. Similarly, clinicians indicated that Feelee gave valuable insights into the adolescents’ emotional needs, thereby enhancing their understanding of the treatment process [65]. Therefore, the incorporation of smartphone data into treatment has the potential to positively influence treatment engagements. Future research should explore the role of Feelee in promoting treatment engagement, particularly during the early stages of treatment and periods of stagnation.

Finally, both adolescents and clinicians offered several recommendations for the further development and application of Feelee in treatment settings. Engaging intended end users is a critical step in the development of effective and valuable mobile health interventions [66]. The engagement facilitates the integration of scientific research with practical application and supports the successful implementation of these interventions in clinical practice [67]. Accordingly, the feedback and suggestions provided by adolescents and clinicians will be instrumental in guiding the design of an updated version of Feelee.

### Limitations

Our study has several limitations. First, the validity of our findings may be compromised by the small sample size of adolescents, which consisted exclusively of male participants. This limitation is partly due to the forensic population being difficult to reach, as these adolescents are often not motivated to engage in treatment or research. As a result, the recruitment of participants was significantly reliant on the collaboration of clinicians, which introduces a degree of selection bias into this study. In addition, the fact that Feelee was only compatible with Android operating smartphones further restricted recruitment, excluding potential participants who used iOS devices. Consequently, these factors together limit the generalizability of our results. Second, the adolescents who participated in this study had already been undergoing treatment for some time before their involvement, potentially increasing their motivation to participate. As a result, these participants may not be fully representative of the broader forensic adolescent population. To enhance the generalizability of findings, future research should aim to include a more diverse sample of adolescents at various stages of treatment. Third, the 2-week test period may be considered insufficient for a comprehensive evaluation of Feelee’s feasibility and usability in the forensic outpatient setting. However, this study served as an initial exploration of Feelee as an innovation whose effects are still largely unknown. Consequently, we restricted the test period for adolescents to a minimum of 2 weeks, in line with the findings from several prepilot studies, to obtain an initial low-burden assessment of the application’s feasibility and usability [68-70]. Future research should ensure that the intervention lasts at least 4 weeks to get a better understanding of its long-term usability and effects. Fourth, the use of semistructured interviews conducted after the test period provided insights only into participants' subjective experiences and perceptions of the app, rather than quantifiable emotional and behavioral changes resulting from app usage. While these interviews offer valuable perspectives on adolescents’ and clinicians’ experiences, they do not fully capture the app’s impact or effectiveness in treatment. Therefore, future studies should incorporate quantitative measures to gain a more comprehensive understanding of Feelee’s effects and operational mechanisms for adolescents in the forensic outpatient setting [66,71].

### Conclusions

This explorative, qualitative, feasibility and usability study provides an initial, in-depth understanding of adolescents’ and clinicians’ experiences with the integration of active and passive smartphone data into treatments within the forensic outpatient setting. The findings from the 2-week test period and semistructured interviews indicate that, despite some technical shortcomings, Feelee was regarded as a valuable addition for enhancing emotional and behavioral insights. Nevertheless, it did not significantly contribute to increasing treatment motivation or strengthening the treatment alliance. While this study offers important perspectives on the feasibility and usability of Feelee, it does not fully assess its impact on emotional or behavioral changes. Furthermore, limitations related to the recruitment process and the small, homogeneous, but motivated sample size may influence the study’s results. Consequently, further research is required to evaluate Feelee’s clinical effectiveness and mechanism for enhancing emotional awareness across a broader forensic population. Future investigations should extend the intervention period, distinguish between different treatment phases, and incorporate quantitative methods to provide a comprehensive evaluation of its effects. This study represents a pioneering effort to demonstrate the feasibility and usability of incorporating smartphone data into forensic treatment. Insights from adolescents and clinicians will be instrumental in refining Feelee and advancing future research in this field.

## Abbreviations

CBT: cognitive behavioral therapy
GDPR: General Data Protection Regulation

## Data Availability

The datasets generated and/or analyzed during the study are available from the corresponding author on reasonable request.
